# Crosstalk Between Trophoblasts and Decidual Immune Cells: The Cornerstone of Maternal-Fetal Immunotolerance

**DOI:** 10.3389/fimmu.2021.642392

**Published:** 2021-02-25

**Authors:** Ling Xu, Yanhong Li, Yifei Sang, Da-Jin Li, Meirong Du

**Affiliations:** ^1^Laboratory for Reproductive Immunology, NHC Key Lab of Reproduction Regulation (Shanghai Institute of Planned Parenthood Research), Shanghai Key Laboratory of Female Reproductive Endocrine Related Diseases, Hospital of Obstetrics and Gynecology, Fudan University Shanghai Medical College, Shanghai, China; ^2^Department of Obstetrics and Gynecology, Guangzhou First People's Hospital, School of Medicine, South China University of Technology, Guangzhou, China

**Keywords:** trophoblasts, decidual immune cells, maternal-fetal immunotolerance, recurrent spontaneous abortion, immunotherapy

## Abstract

The success of pregnancy relies on the fine adjustment of the maternal immune system to tolerate the allogeneic fetus. Trophoblasts carrying paternal antigens are the only fetal-derived cells that come into direct contact with the maternal immune cells at the maternal–fetal interface. The crosstalk between trophoblasts and decidual immune cells (DICs) via cell–cell direct interaction and soluble factors such as chemokines and cytokines is a core event contributing to the unique immunotolerant microenvironment. Abnormal trophoblasts–DICs crosstalk can lead to dysregulated immune situations, which is well known to be a potential cause of a series of pregnancy complications including recurrent spontaneous abortion (RSA), which is the most common one. Immunotherapy has been applied to RSA. However, its development has been far less rapid or mature than that of cancer immunotherapy. Elucidating the mechanism of maternal–fetal immune tolerance, the theoretical basis for RSA immunotherapy, not only helps to understand the establishment and maintenance of normal pregnancy but also provides new therapeutic strategies and promotes the progress of immunotherapy against pregnancy-related diseases caused by disrupted immunotolerance. In this review, we focus on recent progress in the maternal–fetal immune tolerance mediated by trophoblasts–DICs crosstalk and clinical application of immunotherapy in RSA. Advancement in this area will further accelerate the basic research and clinical transformation of reproductive immunity and tumor immunity.

## Introduction

A new life begins when an egg and a sperm meet at the ampulla of the mother's fallopian tube and combine to form a fertilized egg, which further develops into a blastocyst. A series of processes including implantation, decidualization, and trophoblast differentiation and invasion ultimately lead to successful placentation and embryo development (1, 2). Immunologically speaking, either the embryo or the trophoblast carrying paternal antigens is similar to a semi-allogeneic transplant for the mother. However, instead of being attacked by the maternal immune system, the embryo or fetus grows naturally and safely in the womb until delivery. Hence, successful pregnancy is considered to be an immunological paradox that challenges the basic principles of transplantation immunology.

For decades, many researchers have devoted themselves to exploring the immunological truth behind pregnancy. The first attempt to explain the immune refutation was made by famous transplant scientist Peter Medawar in the 1950s (3). He proposed three possible theories, namely, the presence of the placental barrier, the immaturity of fetal antigens, and the inertness of the maternal immune system, which had greatly promoted the development of reproductive immunology at that time and still have important guiding significance nowadays. After years of research, it has been discovered that the complex and delicate dialogs between trophoblasts and decidual immune cells (DICs) are the key link in driving the establishment and maintenance of maternal–fetal immunotolerance. Trophoblasts are the only fetal-derived cells that come into close contact with the mother's immune system. Moderate proliferation, migration and invasion of trophoblasts in early pregnancy are crucial to placenta formation and fetal growth, and are regulated by multiple factors (4, 5). Dysfunction of trophoblast cells may lead to a series of pregnancy-related complications including RSA and preeclampsia (PE) (6, 7). On the other hand, DICs refer to a large number of immune cells accumulating in the decidua during early pregnancy which account for approximately 40–50% of decidual cells (8, 9). Among them, decidual NK cells, decidual macrophages and decidual T cells occupy the vast majority, and the rest include granulocytes, dendritic cells, mast cells, other innate lymphoid cells (ILCs), decidual B cells and so on (8, 10). DICs have specific phenotypes and are involved in the regulation of crucial processes including local inflammation and immune responses, trophoblast invasion and vascular remodeling (8). Actually, the phenotypes and functions of DICs can be finely adjusted by trophoblasts, the active builder of immune tolerance in pregnancy. Aberrant interaction between trophoblasts and DICs will cause immune tolerance disorder, which is closely related to a variety of adverse pregnancy outcomes such as recurrent spontaneous abortion (RSA), intrauterine growth retardation (IUGR), and PE (11). Therefore, this review will elaborate on the complicated and delicate interaction between trophoblasts and DICs in the formation of maternal–fetal immunotolerance, in the hope of providing new ideas for the diagnosis and treatment of clinical pregnancy-related diseases.

## The Differentiation and Characteristics of Trophoblasts

**Figure 1:**
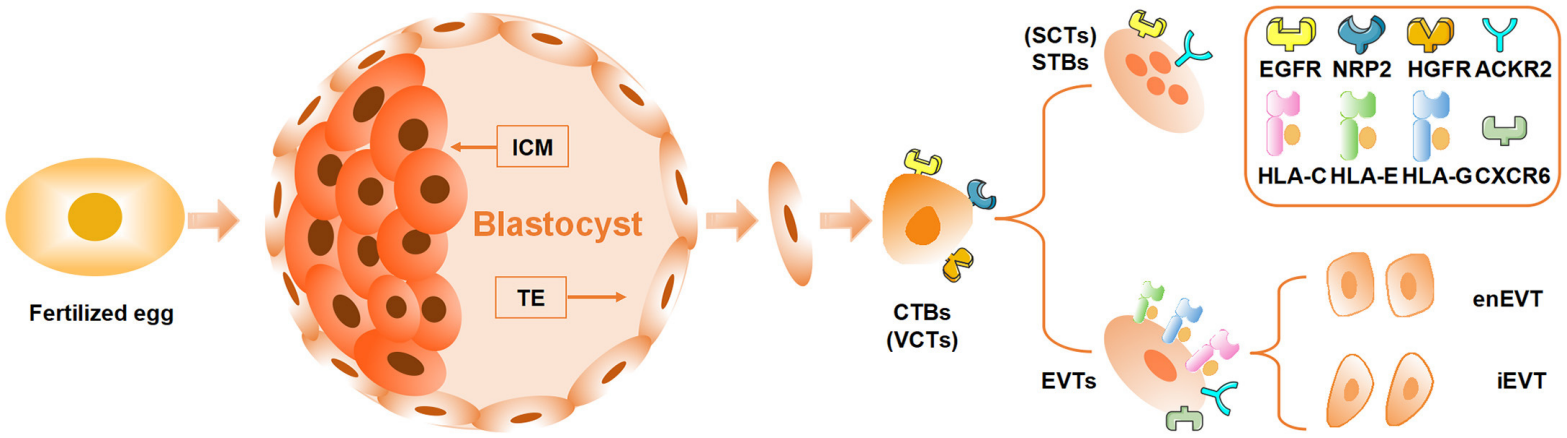
Differentiation and development of trophoblasts. The blastocyst developed from the fertilized egg further differentiates into inner cell mass (ICM) and trophectoderm (TE). ICM develops into the fetus, while TE differentiates into cytotrophoblasts (CTBs or villous cytotrophoblasts, VCTs) which further differentiate into syncytiotrophoblasts (STBs or SCTs) and extravillous trophoblasts (EVTs). The EVTs that invade the decidua can be divided into intravascular EVTs (enEVTs) and interstitial EVTs (iEVTs) according to the specific location. EGFR, epidermal growth factor receptor; NRP2, neuropilin-2; HGFR, hepatocyte growth factor receptor; ACKR2, atypical chemokine receptor 2; HLA, human leukocyte antigen; CXCR6, C-X-C chemokine receptor type 6.

As the only component containing paternal antigens at the maternal–fetal interface, trophoblasts serve a core role in mediating maternal tolerance toward the embryo. It is necessary to have knowledge of the differentiation and characteristics of trophoblasts to fully understand the cross-communication initiated by trophoblasts in maternal immunotolerance. In the pre-implantation stage, the blastocyst differentiates into inner cell mass (ICM) and trophectoderm (TE). After implantation, ICM cells further differentiate into embryonic lineages, giving rise to all kinds of tissues in the developing fetus, whereas TE cells subsequently become the precursor lineage, forming the embryonic part of the placenta (Figure 1) (12). The placenta, a unique and transient organ consisting of both maternal and fetal tissues, not only is in charge of nutrition exchange between the mother and the fetus but also participates in the immune adaptation of the maternal immune system (13, 14). Malfunction of placenta is identified as a potential cause of various pregnancy complications including RSA, PE, and preterm (15, 16). The villus, the functional unit of placenta, is composed of epithelial trophoblasts differentiated from TE and a stromal cell core containing fetal endothelial cells, Hofbauer cells, and mesenchymal stromal cells, among others (17). Cytotrophoblasts (CTBs), one type of epithelial trophoblasts, encircle the stromal cell core and express receptors involved in cellular proliferation and differentiation such as epidermal growth factor receptor(EGFR), neuropilin-2 (NRP2), and hepatocyte growth factor receptor(HGFR) which are predicted to interact with Hofbauer cells and placental fibroblast cells (Figure 1, Table 1) (18). There are two distinct differentiation pathways in CTBs, generating syncytiotrophoblasts (STBs) and extravillous trophoblasts (EVTs), the other two types of epithelial trophoblasts (Figure 1) (18). STBs, responsible for producing placental hormones, scarcely express any major histocompatibility complex (MHC, also known as human leukocyte antigen [HLA]) class I or class II molecules, which allows them to escape immune attack mediated by allogeneic recognition from T cells (19). By contrast, EVTs, which are in charge of invading the decidua and participating in spiral arteries remodeling, express HLA-C, HLA-E, and HLA-G along with increased expression of receptors involved in immune regulation including atypical chemokine receptor 2 (ACKR2) and C-X-C chemokine receptor type 6 (CXCR6) (Figure 1, Table 1) (18).

**Table 1:** New subsets of placental trophoblasts during early pregnancy identified by single cell RNA seq.

	**CTB**	**EVT**
Subsets	Proliferative (Replenishment of the CBT pool)	RRM2^+^ (cell cycle and division) The proximal end of cell column
	Non-proliferative	RRM2^low/−^ The distal end of cell column
Receptors	EGFR	HLA-C
	NRP2	HLA-E
	HGFR	HLA-G
		ACKR2
		CXCR6
Functions	Proliferation	Immunomodulation
	Differentiation	Invasion

*There are three CTB subsets present in the first trimester placenta according to cell cycle analysis: the proliferative subset and other two non- proliferative subsets (17). Based on the expression level of RRM2 EVT can be divided into three subtypes: the positive one located at the proximal end of cell column and the other two subtypes with little or low expression of RRM2 which locates at the distal end of cell column (17). CTB, cytotrophoblast; EVT, extravillous trophoblast; EGFR, epidermal growth factor receptor; NRP2, neuropilin-2; HGFR, hepatocyte growth factor receptor; RRM2, ribonucleotide reductase regulatory subunit M2; HLA, human leukocyte antigen; ACKR2, atypical chemokine receptor 2; CXCR6, C-X-C chemokine receptor type 6*.

Recently, single-cell RNA sequencing performed on placental cells has identified three subsets of CTBs. The proliferative subpopulation was predicted to replenish the CTB pool. The non-proliferative subset was divided into two other subgroups on the basis of the expression of Syncytin-2, of which the positive one was proved to be STB progenitors (Table 1) (17). Not only that, EVTs from the first trimester were also identified as three subsets, and EVT subsets expressed more than 40 polypeptide hormone genes including CSH1, FSTL1, PAPPA2, TAC3, and several PSG genes. This is contradictory to previous cognition that other placental subsets hardly secret hormones except STBs (Table 1) (17). New insights on the phenotypes and functions of various types of placental cells have greatly enriched and deepened our understanding of the human placental lineage specification and deserve further research in the future. For example, do these new hormone genes expressed by EVT subsets also play a role in the induction of immune tolerance? This is a new scientific question waiting to be answered.

## The Formation of Maternal-Fetal Immunotolerance

**Figure 2:**
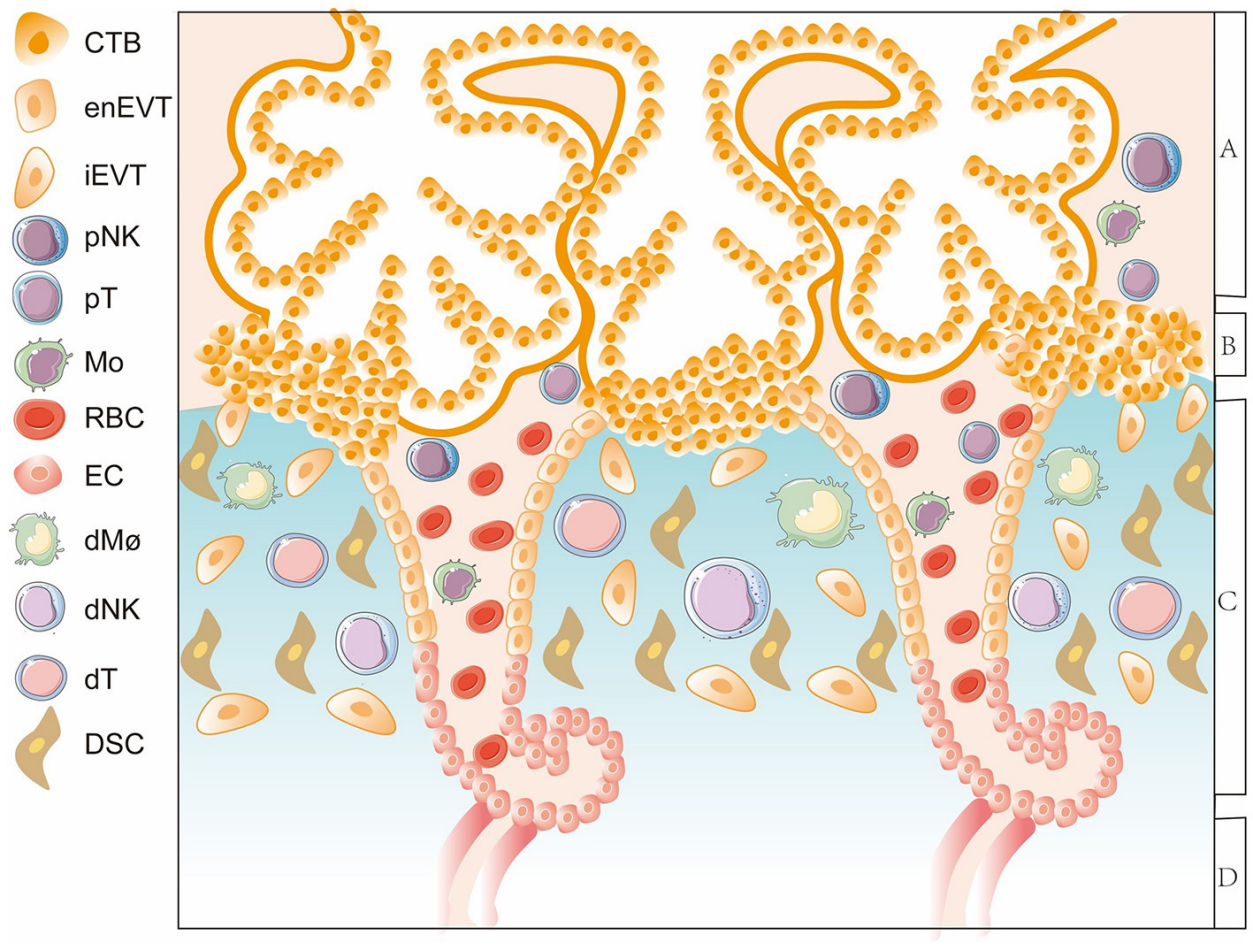
A diagram of the maternal-fetal interface in the first trimester. The thick lines surrounding CTBs represent STBs. Area A indicates the floating villi and interstitial spaces. Area B represents the villous column formed by trophoblasts. Area C represents the decidua layer. Area D indicates the myometrium. The important maternal-fetal interface is composed of EVTs, DSCs, and decidual immune cells. CTB, cytotrophoblast; enEVT, endovascular EVT; iEVT, interstitial EVT; pNK, peripheral natural killer cell; pT, peripheral T cell; Mo, monocyte; RBC, red blood cell; EC, endothelial cell; dMΦ, decidual macrophages; dNK, decidual natural killer cell; dT, decidual T cel; DSC, decidual stromal cell.

Trophoblasts communicate with decidual cells in specific microenvironments, namely, the important maternal–fetal interface which appears in early pregnancy when EVTs invade the decidua and encounter various DICs as well as a large number of decidual stromal cells (DSCs) (Figure 2) (20). The maternal–fetal interface is the key site to establish a specific immune microenvironment and maintain maternal–fetal immune tolerance. Various types of DICs greatly influenced by EVTs are characterized with specific phenotypes and functions. Decidual natural killer (dNK) cells are the most abundant, reaching more than 70% of decidual lymphocytes, followed by decidual macrophages (dMΦ) with 10–20% and then decidual T cells, which make up 10–20% of total DICs (Figure 2) (21). EVTs actively engage in precise dialogs with DICs to tolerate the allogeneic fetus (22). However, aberrant crosstalk between EVTs and DICs leads to dysregulated immune responses and imbalanced maternal–fetal immune tolerance, which can bring about a series of pregnancy complications including RSA, the most common event occurred in early pregnancy (9). In the following sections, we will elaborate the characteristics of immune cell subgroups and their intricate interaction with trophoblasts to conduce pregnancy tolerance, depicting the network of maternal–fetal immunity.

### Decidual NK Cells

#### A Brief Description of the Phenotype and Characteristics of dNK Cells

NK cells, as important members of the innate immune system, are involved in antiviral and anti-tumor immunity. In general, peripheral blood NK (pNK) cells account for about 10% of total lymphocytes, and the phenotype of the vast majority (90–95%) is CD3^−^CD56^dim^CD16^+^, representing stronger cytotoxicity, whereas the remaining 10% is CD3^−^CD56^bright^CD16^−^, mainly secreting various cytokines. This shows a distinct phenotype of dNK cells characterized by CD3^−^CD56^bright^CD16^−^CD9^+^KIR^+^ from that of pNK cells, which also indicates that the local microenvironment of the decidua is closely related to the differentiation of dNK cells (23).

#### Interaction Between Trophoblasts and dNK Cells in Maternal–Fetal Tolerance

**Figure 3:**
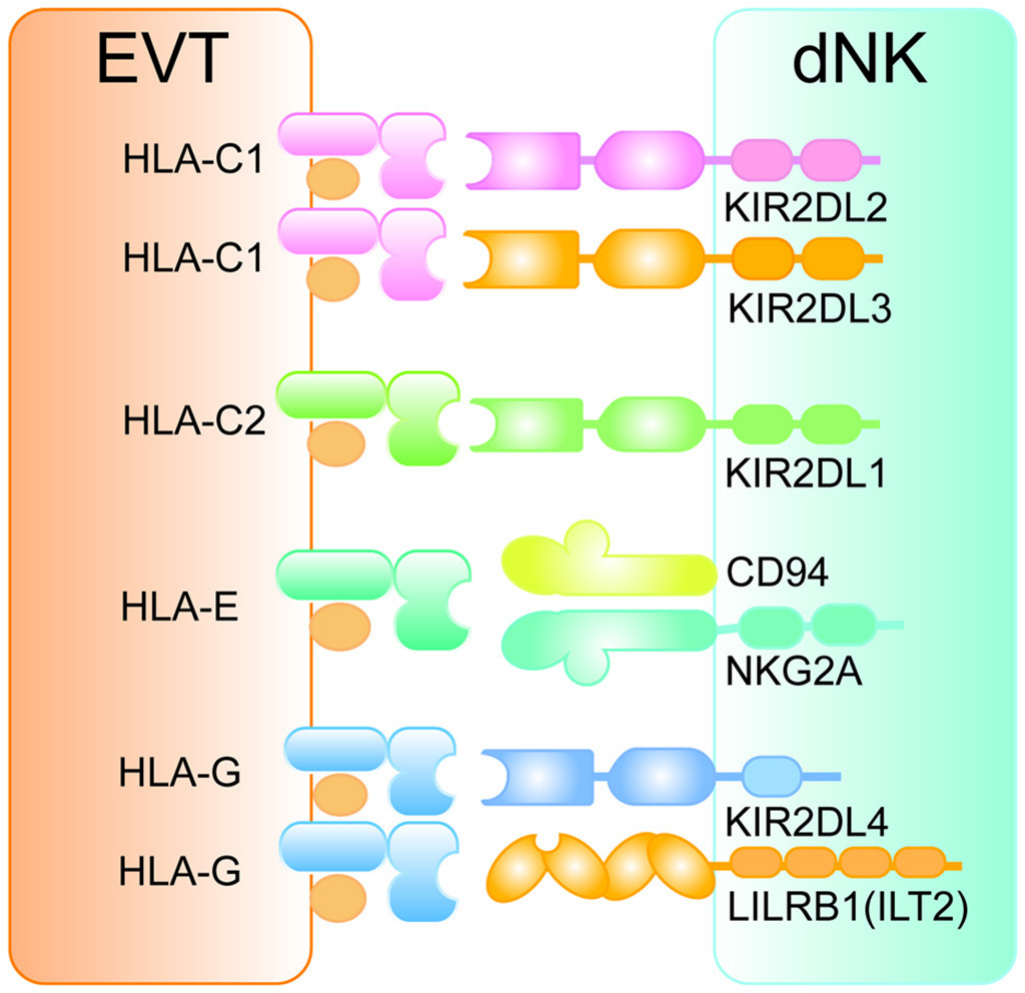
The interaction between HLA ligands on EVTs and inhibitory receptors on dNK cells. The pattern diagram shows the expression profile of HLA antigens on EVTs and the corresponding inhibitory receptors on dNK cells. All inhibitory receptors contain at least one ITIM motif in their intracellular regions. EVT, extravillous trophoblasts; HLA, human leukocyte antigen; dNK, decidual natural killer cell; KIR, killer cell immunoglobulin receptor; LILRB1, leukocyte immunoglobulin-like receptor subfamily B member 1; ILT2, immunoglobulin-like transcript 2.

Whether peripherally or locally, execution of cytotoxic and/or cytokine secretion functions of NK cells is dependent on the recognition of MHC ligands by their membrane surface receptor repertoire (24). Human trophoblasts, as the only cell type carrying paternal antigens at the mother–fetus interface, express a unique repertoire of MHC ligands. The MHC ligands HLA-C, HLA-E, and HLA-G expressed by EVTs are the targets of dNK cells (Figure 3). The surface receptors of dNK cells can be divided into activating receptors and inhibitory receptors. To be exact, the activation or inhibition of dNK cells hinges on the binding ability of the inhibitory receptors with corresponding MHC ligands expressed by EVTs, indicating a crucial role of EVTs on regulation of dNK cell functions (22). In humans, HLA-C can be classified into two allotypes due to its dimorphism, HLA-C1 and HLA-C2 (25). The corresponding receptors expressed on dNK cells are killer cell immunoglobulin receptors (KIRs), which include inhibitory receptors (KIR2DL2 or KIR2DL3 specific for HLA-C1 and KIR2DL1 specific for HLA-C2) and activating receptors (KIR2DS1 specific for HLA-C2) (Figure 3) (26). On the basis of the presence of activating receptors, hundreds of KIR genotypes can be divided into two haplotypes, A (inhibitory receptors only) and B (activating and inhibitory receptors) (26). Although the combination of KIR inhibitory receptors with HLA-C is indispensable for dNK cells to recognize and tolerate fetal antigens, the lack of appropriate activation of dNK cell function mediated by KIR activating receptors may lead to adverse pregnancy outcomes such as IUGR, PE, and RSA (27–29).

In addition to the allogeneic recognition of HLA-C with KIRs, the interaction between HLA-E and CD94/NKG2 receptors on dNK cells is also one of the basic mechanisms regulating its activity (Figure 3). Here is a brief introduction to the CD94/NKG2 receptors library. Genes including *CD94* (*KLRD1*), *NKG2D* (*KLRK1*), *NKG2F* (*KLRC4*), *NKG2E* (*KLRC3*, encoding NKG2E and NKG2H), *NKG2C* (*KLRC2*), and *NKG2A* (*KLRC1*, encoding NKG2A and NKG2B) are grouped into a cluster (30). Except NKG2D and NKG2F, both of which are activating receptors, the CD94 molecule generally forms a heterodimer transmitting inhibitory signals with NKG2A/NKG2B and a heterodimer having activating functionality with NKG2C/NKG2E/NKG2H (30). Engagement of CD94/NKG2A with HLA-E expressed by EVTs (Figure 3) inducing inhibitory signals is an essential mechanism by which dNK cells maintain tolerance to the semi-allogeneic fetus (31).

HLA-G expressed by EVTs regulates the activity of dNK cells in several ways. On the one hand, HLA-G can directly bind inhibitory receptors KIR2DL4 and Immunoglobulin-like transcript 2 (ILT2, also known as leukocyte immunoglobulin-like receptor subfamily B member 1 [LILRB1]) on dNK cells (Figure 3) (32, 33) or indirectly enhance the affinity between inhibitory receptor CD94/NKGA and HLA-C (34). On the other hand, HLA-G-induced immunotolerance can be achieved by a special biological process, termed trogocytosis, which means transfer of membrane proteins between cells. Successful HLA-G trogocytosis endows the recipient cells with a transient immunosuppressive phenotype (35). HLA-G can be captured via trogocytosis and internalized via endocytosis by dNK cells during interaction of HLA-G^+^ EVTs with dNK cells. Degradation of internalized HLA-G occurs after cytokine activation and is accompanied with restoration of cytotoxicity, suggesting trogocytosis of HLA-G is closely related to the tolerant phenotype of dNK cells (36).

In addition to specific HLA molecules, trophoblasts can help to maintain the unique phenotype and restrained activity of dNK cells in other ways. Our team previously found that trophoblasts-derived galectin-9 interacting with T cell immunoglobulin mucin receptor 3 (Tim-3) on pNK cells promoted a shift toward the dNK cell-like phenotype. Moreover, trophoblasts limited excess activation of dNK cells evoked by lipopolysaccharide (LPS) stimulation in a galectin-9/Tim-3 dependent manner (37). All the results suggest that the checkpoint Tim-3-mediated signaling plays a role in the trophoblasts–dNK cells communication. Other researchers implied that the autophagy level of trophoblasts is bound up with the cytotoxicity of dNK cells. Tan et al. showed that trophoblasts with upregulated autophagy levels induced by rapamycin greatly inhibited the expression of CD16 and activating receptors such as NKG2D, NKp30, and NKp46 on dNK cells via IGF2 signaling (38). Besides, indirect regulation of dNK cells by trophoblasts was also proved. Our group demonstrated that C-X-C motif chemokine ligand 16 (CXCL16) secreted by trophoblasts induced M2 macrophage polarization and the instructed M2 facilitated the inactivation of NK cells via the decreased expression of interleukin (IL)-15 (39).

Recently, an interesting result of the transcriptome analysis of single cells from first-trimester pregnancy has attracted most attention. Analysis revealed that there were three main subgroups of dNK cells: dNK1, dNK2, and dNK3 cells. The same subgroup classification was also characterized in another study using mass cytometry (40). Compared with dNK2 and dNK3 cells, dNK1 cells expressed higher levels of KIRs and LILRB1, which are inhibitory receptors for HLA-C and HLA-G, respectively, as mentioned above, and produced more perforin and granzyme B (18). The specific inhibitory receptor CD94/NKG2A for HLA-E was expressed by both dNK1 and dNK2 cells (18). This finding suggests that dNK1, rather than dNK2 or dNK3, may be the main subgroup that recognizes and interacts with EVTs.

Whether it is the novel subpopulations of placental cells mentioned above or the breakthrough new classification of decidual NK cells summarized here, researchers benefited from single-cell sequencing, one of the most popular research techniques, have been able to discover something new from what they already know. However, it is worth noting that although single-cell sequencing can provide rich biological information for comprehensive and in-depth researches, the existence and difference of mRNA levels do not necessarily mean the same changes in protein levels. In short, the results of single-cell sequencing are not guaranteed to be completely reliable, but they can indeed provide hints or guidance for future research directions.

In summary, as the most abundant immune cells in the decidua during early pregnancy, the specific phenotype and function of NK cells require the fine adjustment of the local microenvironment of the decidua, including trophoblasts. However, it should be reminded that there is no guarantee that decidual NK cells will always play a friendly role in pregnancy. When the microenvironment is abnormal, such as in the case of inflammation, NK cells may become adversely activated, which not only fails to be the guardian of the fetus, but becomes the foe to pregnancy (41–43).

### Decidual Macrophages

#### Dynamic M1/M2 Shift of Decidual Macrophages

Macrophages, the pivotal regulators and effectors of inflammation and innate immune responses (44), are the immune cells with the highest plasticity in the hematopoietic system. They are distributed in all tissues and play important roles in almost all kinds of biological processes of the body, both physiologically and pathologically (45). Tissue-resident macrophages derive from a wide range of sources, including (1) bone marrow-derived monocytes, (2) embryonic progenitor located in the yolk sac or fetal liver, and (3) self-renewal (46). In response to specific microenvironmental stimuli, macrophages undergo specific functional polarization, resulting in classically activated M1 macrophages and alternatively activated M2 macrophages (47). M1 macrophages characterized by CD80 and CD86 are supposed to participate in antigen presentation and secretion of proinflammatory cytokines, whereas M2 macrophages with surface markers CD206 and CD163 take charge of tissue remodeling and immune tolerance mediated by Th2 activation (48). *In vitro*, monocytes are induced to polarize toward the M1 phenotype with LPS and IFN-γ and toward the M2 phenotype with IL-4 and IL-13 (45). Disturbed M1/M2 macrophages balance at the maternal-fetal interface was proved to participate in the adverse pregnancy outcome (49, 50). Excessive activation of decidual macrophages displaying higher expression of pro-inflammatory cytokines and lower anti-inflammatory cytokines was detected in patients with recurrent miscarriage (49, 51, 52).

At different stages of pregnancy, the maternal immune system presents different inflammatory states. In the beginning, a moderate inflammatory environment is conducive to embryo implantation. Then, the local decidua needs to establish an anti-inflammatory and immune-tolerant microenvironment to ensure the survival and growth of the embryo. At the time of delivery, the microenvironment of the decidua shifts toward the proinflammatory direction again (53). The phenotype and function of macrophages at the maternal–fetal interface change accordingly. Before implantation, dMΦ polarize toward the proinflammatory M1 phenotype. When EVTs begin to invade the decidua after implantation, a mixed M1/M2 profile is present and gradually transforms into a tolerant M2 phenotype until delivery (21). Indeed, single-cell sequencing confirmed M1 and M2 macrophages coexisting at the maternal–fetal interface during the first trimester (18). Notably, it is previously agreed that decidual M2 macrophages highly express IL-10, an immune regulatory cytokine that plays a crucial role in immunotolerance, but the result of single-cell RNA sequencing revealed a higher expression level of IL-10 in decidual M1 macrophages than that in decidual M2 macrophages (18). Because IL-10 itself is one of the stimuli inducing M2 polarization, there is a possibility, which remains to be confirmed, that decidual M1 macrophages promote their own transformation to the tolerant M2 phenotype via the secretion of IL-10. What is clear, however, is that the predominance of decidual M2 macrophages contributes to the maintenance of an immunotolerant environment.

#### The Conversation Between Trophoblasts-dMΦ in Promoting M2 Polarization Shift

**Figure 4:**
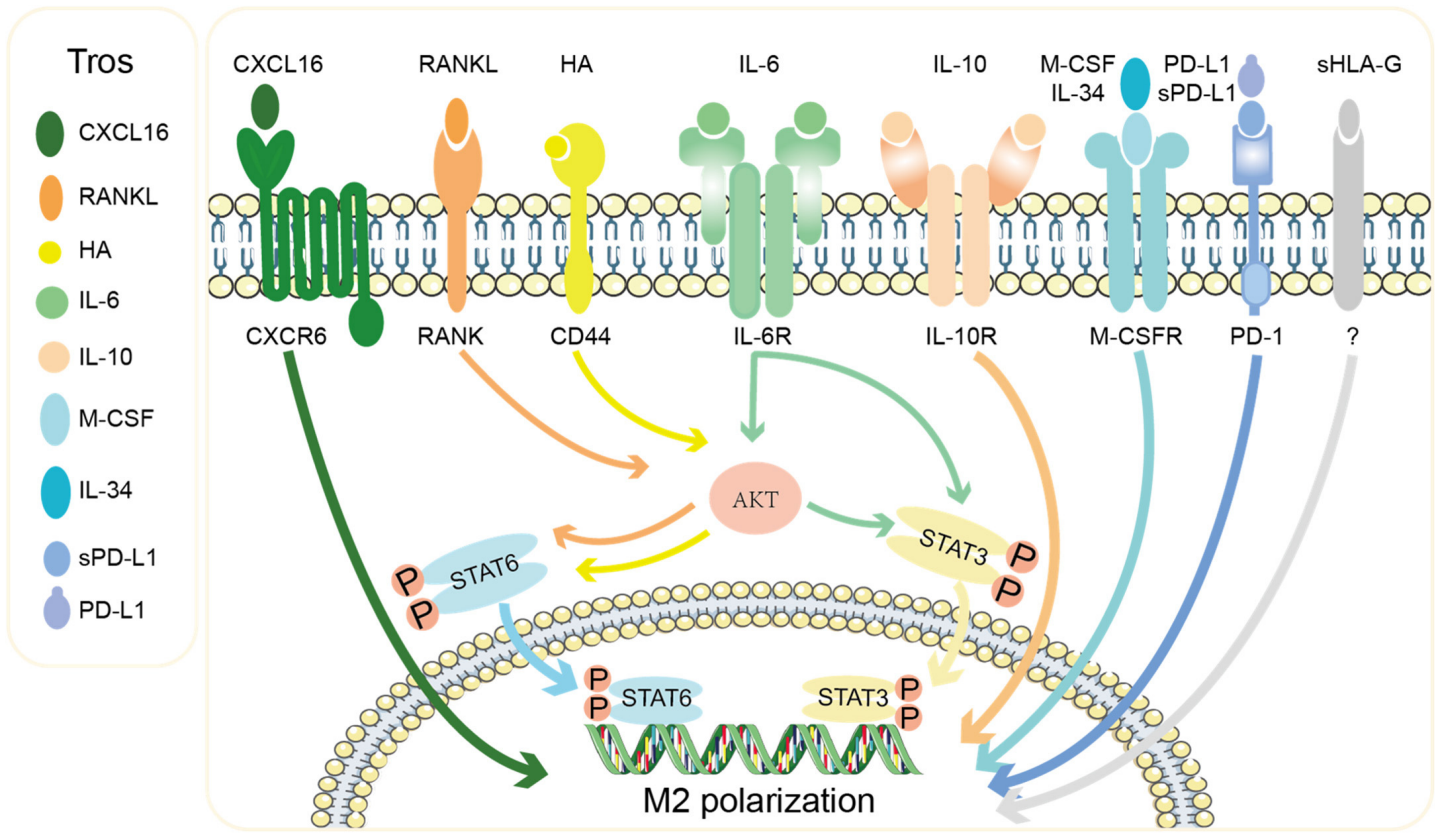
Mechanisms of the M2 polarization of decidual macrophages by trophoblasts. Tros express chemokine CXCL16, extracellular matrix HA, cytokines (RANKL, IL-6, IL-10, M-CSF, and IL-34), and checkpoint ligands PD-L1 and sPD-L1, which act on macrophages to promote them to polarize toward M2 phenotype via different signal pathways. Tros, trohoblasts; CXCL16, C-X-C motif chemokine ligand 16; RANKL, receptor activator for nuclear factor-κ B ligand; HA, hyaluronic acid; IL, interleukin; M-CSF, macrophages colony stimulating factor; sPD-L1, soluble programmed cell death 1 ligand 1; sHLA-G, soluble human leukocyte antigen G; CXCR6, C-X-C chemokine receptor type 6; RANK, receptor activator for nuclear factor-κ B; STAT, signal transducer and activator of transcription.

Numerous studies have shown that trophoblasts zealously promote the predominant M2 polarization of dMΦ in multiple ways to maintain an anti-inflammatory and immune-tolerant environment (Figure 4). Interesting findings reported by Abumaree et al. (54) that the phagocytosis of trophoblast debris by macrophages led to increased expression of programmed cell death 1 ligand 1 (PD-L1) and IL-10, as well as decreased expression of costimulatory molecules (CD80, CD86, CD40, and B7H3) and proinflammatory cytokines including IL-1β, IL12p70, and IL-8. Our team has demonstrated that the chemokine CXCL16 secreted by trophoblasts recruited peripheral monocytes to the decidua by interacting with the receptor CXCR6 (55) and promoted the polarization of macrophages into the M2 phenotype which exhibit decreased IL-15 production, so as to facilitate the inactivation of NK cells (39). We also found that the receptor activator for nuclear factor-κ B ligand (RANKL), expressed by DSCs and in particular trophoblasts, interacted with RANK on dMΦ and activated the downstream AKT/signal transducer and activator of transcription (STAT)-6 signaling, inducing M2 polarization, which further induced CD4^+^ T cells to present a Th2 bias (56). Placenta-derived soluble factors macrophage colony stimulating factor (M-CSF, also known as CSF-1) and, in particular, IL-10, both of which were primarily produced by trophoblasts, were capable of inducing homeostatic M2 macrophages characterized by the decidual-like CD14^+^CD163^+^CD206^+^CD209^+^ phenotype and production of IL-10 and CCL18 (57). Higher level of IL-10 is produced by placental villous trophoblasts during first and second trimesters. And it has been demonstrated that in both human and mouse, IL-10 exerts anti-inflammatory and immunosuppressive effect on multiple decidual immune cells including macrophages and mediates the immune tolerance in pregnancy (58). Observations in IL-10^−/−^ mouse suggest that IL-10 deficiency is strongly associated with adverse pregnancy outcomes including RSA, PE, and preterm delivery (42, 59–61). Besides, IL-34, a newly discovered cytokine sharing the same receptor as M-CSF, was produced by trophoblasts and DSCs and could polarize macrophages into a decidual-like phenotype *in vitro* (62). Recently, Ding et al. (63) found that trophoblasts skewed macrophages toward M2 polarization via secreting IL-6 that activated signal transducer and activator of transcription (STAT)-3 signal in a co-culture system. In addition to chemokines and cytokines, trophoblasts could also secrete hyaluronic acid, the most abundant component of the extracellular matrix, which interacted with CD44 and activated the downstream PI3K/Akt-STAT-3/STAT-6 signal pathway to promote M2 polarization (Figure 4) (64).

The interaction of checkpoint PD-1 and its ligand PD-L1 has also been reported to involve in decidual M2 polarization (Figure 4). Zhang et al. (65) showed that trophoblasts expressed PD-L1 and activated PD-1 on dMΦ, conducing to the M2 phenotype with immunomodulatory characteristics. Recently, soluble PD-L1 (sPD-L1) was found to be elevated throughout gestation and predicted to suppress maternal immune responses (66, 67). A research found that sPD-L1 produced by trophoblasts could induce unique CD14^+^CD206^+^CD86^−^ macrophages, which expressed IFN-β in response to TLR4/LPS activation. IFN-β produced by the induced macrophages in turn promoted the secretion of sPD-L1 by trophoblasts (68). Similar to sPD-L1, trophoblast-produced soluble HLA-G5, translated from one of seven alternatively spliced transcripts of HLA-G, drove the differentiation of macrophages toward an immunoregulatory phenotype accompanied by increased expression of indoleamine 2,3-dioxygenase (IDO) 1 and IL-6, both of which are closely related with the inhibition of T cell function (69).

#### Decidual T Cells

As the main adaptive immune cells at the maternal–fetal interface, decidual T cells have always been one of the hotspots in the study of reproductive immunity. The number and functions of decidual T cells are precisely regulated in both human and mice pregnancy, and their abnormalities or dysfunction may lead to many pregnancy diseases such as miscarriage and PE (70–72). Decidual T cells mainly include two cell subgroups: CD4^+^ T cells, accounting for 30–45%, and CD8^+^ T cells, making up 45–75%. According to cytokine production and different transcription factor requirements, CD4^+^ T cells are further divided into Th1, Th2, Th17, and regulatory T (Treg) cells, whose proportions of total decidual CD4^+^ T cells are 5–30, 5, 2, and 5%, respectively (21). However, some studies have shown that the heterogeneous subpopulations of CD4^+^ decidual T cells mainly include Th1, Th17 and Treg subsets, since the mRNA levels of Th2-type cytokines (*IL4, IL5*, and *IL13*) and *GATA3* at rest as well as the secretion level of IL4 after stimulation are extremely low in CD4^+^ decidual T cells (73).

#### Crosstalk Between Trophoblasts and Decidual Effector T Cells Facilitates Maternal Tolerance

Th1 cells, whose essential transcription factor is T-bet, are differentiated from naïve CD4^+^T cells in response to IL-12 and produce Th1-type cytokines including IL-2, IFN-γ, and TNF-α, which are associated with cellular immunity and immune rejection (74, 75). In contrast, Th2 cells characterized by transcription factor GATA-3 are induced by IL-4 and responsible for secreting Th2-type cytokines such as IL-4, IL-5, IL-6, IL-10, and IL-13 (74, 76). Th17 cells, whose specific transcription factor is RORγt, are another effector T subset in response to IL-6 and transforming growth factor-β (TGF-β), secreting proinflammatory cytokines such as IL-17A and IL-22 (74, 76).

**Figure 5:**
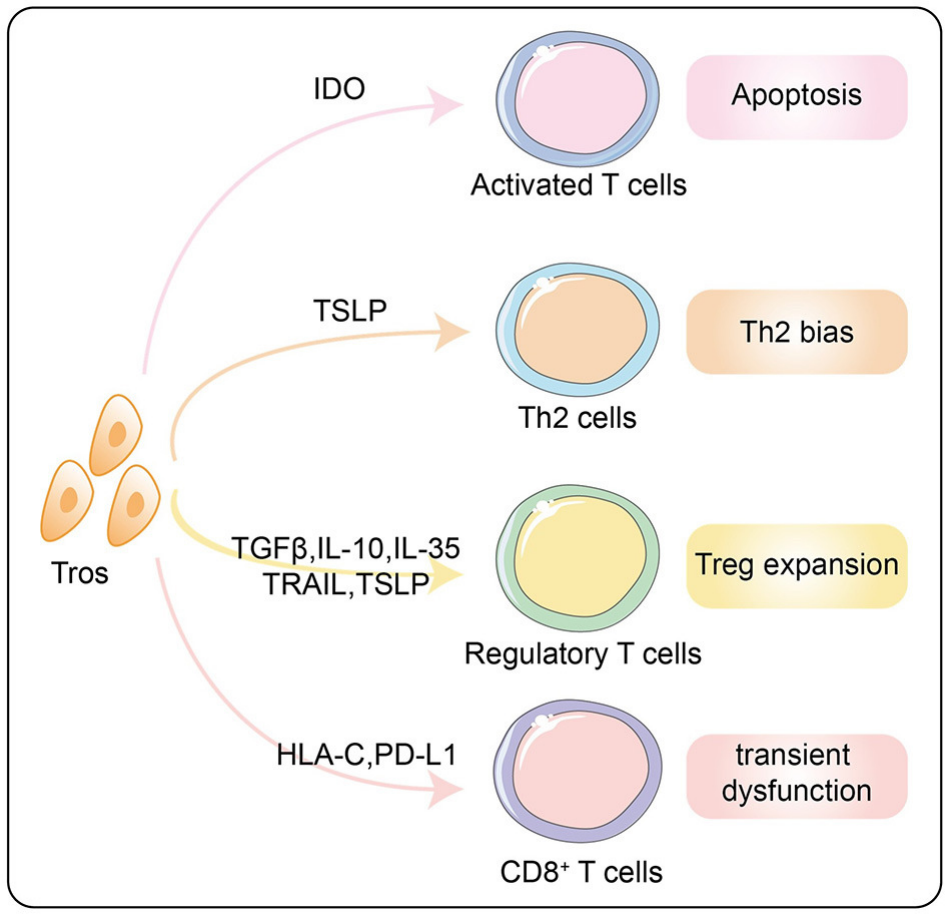
Effects of trophoblasts on decidual T lymphocytes to form the immunotolerance. Tros promote the apoptosis of activated T cells through IDO and inhibit the cytotoxicity of CD8^+^ T cells via HLA-C and PD-L1. Th2 bias and Treg expansion induced by trophoblasts via multiple mechanisms are summarized in this diagram. Tros, trohoblasts; IDO, indoleamine 2,3-dioxyenase; TSLP, thymic stromal lymphopoietins; TGF-β, transforming growth factor-β; IL, interleukin; TRAIL, TNF-related apoptosis-inducing ligand; HLA, human leukocyte antigen; PD-L1, programmed cell death 1 ligand 1.

For the three major effector subgroups of CD4^+^ T cells at the maternal–fetal interface, the attitude of trophoblasts is to shift Th1/Th2 balance toward Th2 bias and suppress Th17 activation, which is in line with the anti-inflammatory and immunotolerant microenvironment required by an allogeneic fetus (77). In fact, trophoblast cells protect themselves from effector T cells attack through a variety of mechanisms. A study published in *Science* revealed that IDO expressed by trophoblasts as well as macrophages inhibited activity of T cells to prevent allogeneic rejection by catabolizing tryptophan (78). Mechanistically, tryptophan deficiency caused cell cycle arrest and apoptosis of activated T cells (79). In addition, our team has proved that the thymic stromal lymphopoietins (TSLPs) secreted by trophoblasts activated CD1c^+^ dendritic cells by binding to the TSLP receptor so as to prime CD4^+^ decidual T cells for Th2 cell differentiation, reflecting the indirect promotion of trophoblasts to Th2 bias (Figure 5) (80). Interestingly, it was found in mice experiments that trophoblasts-derived TSLP could activate placental DC cells through TSLP-TSLP receptor interactions, which in turn promoted the expansion of IL-10^+^NK cell (81).

#### Crosstalk Between Trophoblasts and the Immune-Suppressive Treg Cells

As a unique subset of T cells with immunomodulatory and immunosuppressive properties, Treg cells characterized by CD4^+^CD25^+^FOXP3^+^ regulate all of effector T subsets and are the key hub for the maintenance of T immune homeostasis. The immunosuppressive function of Treg cells is achieved through cell-to-cell contact-dependent and -independent pathways. The key to the former pathway lies in the constitutive expression of immune checkpoint molecule CTLA-4 on Treg cells, which competitively binds to CD80 and CD86 on antigen-presenting cells (APCs) with a higher affinity than CD28 expressed by effector T cells, blocking the indispensable second signal for the activation of T cells (82, 83). IL-2 signals are necessary for the activation of T cells including Treg cells (84). The high affinity IL-2Ra (CD25) on Treg cells not only promotes its own maturity and expansion by receiving IL2 signals produced by effector T cells but also promotes the apoptosis of effector T cells via the consumption of IL-2 (85, 86). While the cell-to-cell contact-independent pathway refers to the secretion of anti-inflammatory and immunosuppressive cytokines such as TGF-β, IL-10, and IL-35 (87, 88), all of which are conversely key regulators of Treg cell expansion and suppressive activity (89–91).

Treg cells can be divided into thymic-derived Treg (tTreg) cells and peripheral Treg (pTreg) cells according to their differentiation and maturation locations. The key to the former is the interactions with self-peptide/MHC class II complexes along with IL2 receptor signaling, while the latter develops from naïve T cell precursors after exposure to antigenic stimulation (92). Besides, a Foxp3 enhancer, conserved noncoding sequence 1 (CNS1), is crucial for pTreg cell differentiation but dispensable for tTreg cell generation (93). It was suggested that Treg cells in decidua were mainly composed of pTreg cells. Deletion of pTreg due to CNS1 knockout showed increased fetal absorption accompanied by increased immune cell infiltration and defective spiral artery remodeling (93, 94). However, a recent study revealed that blunted expansion of tTreg mediated by RANK deficiency in thymic epithelial cells contributed to reduced accumulation of tTreg cells in the placenta and increased fetal loss (95). Increasing evidence has shown that the differentiation and expansion of Treg cells can be commendably adjusted by trophoblasts in multiple ways during pregnancy. First of all, trophoblasts could induce the expansion of CD25^high^CD127^low^Foxp3^+^ Treg cells accompanied by an increased expression of IL-10. This induction was achieved through the cooperation of several placenta-derived soluble factors including transforming growth factor-β (TGF-β), IL-10, and the apoptosis-inducing factor TNF-related apoptosis-inducing ligand (TRAIL) (57). Indeed, it was demonstrated that trophoblasts constitutively expressed a high level of TGF-β, which promoted the recruitment and differentiation of Treg cells (96). Similar result was obtained in another experiment that HLA-G^+^ EVTs co-cultured with matched CD4^+^ T cells led to the augment of CD4^+^CD25^HI^FOXP3^+^CD45RA^+^ resting Treg cells (97). Recently, further research has shown that endovascular extravillous trophoblasts (enEVTs), but not interstitial extravillous trophoblasts (iEVTs), were capable of promoting the differentiation of naïve CD4^+^ T cells into immunosuppressive Treg cells in a TGF-β1-dependent manner, establishing immune tolerance along the placental–maternal circulation (98). As mentioned earlier, IL-35 is also an important regulator of Treg cell function and differentiation. Studies have found that IL-35 secreted by trophoblast cells suppressed T cell proliferation and induced the conversion of naïve conventional T (T_conv_) cells into IL-35-producing induced regulatory T (iT_R_35) cells through the downstream phosphorylation of STAT1 and STAT3 (99). In addition, trophoblasts exerted an indirect induction effect on Treg cell differentiation via first priming antigen presenting cells (APC) decidual dendritic cells (DCs) by the secreted TSLPs (Figure 5) (100). There is a view that a shift from Th17/Treg cell balance toward Treg cell dominance at the maternal–fetal interface is essential for maintaining the immunotolerant microenvironment (75). It was found that the abnormally upregulated CD81 of trophoblasts promoted the differentiation of T cells into Th17 and reduced the differentiation into Treg cells through the paracrine effect of IL-6, which interfered with the immune balance of the maternal–fetal interface and was involved in PE (101).

#### Crosstalk Between Trophoblasts and the Inertial CD8^+^ Decidual T Cell

CD8^+^ decidual T cells are the main force at the maternal–fetal interface to balance the immunotolerance and immune defense (102). Unlike the fact that nearly half of CD8^+^ T cells in peripheral blood are unprimed naïve cells, it is found that CD8^+^ decidual T cells mainly consist of differentiated CD45RA^−^CCR7^−^ effector-memory T cells (103). They express less level of perforin and granzyme B than do peripheral CD8^+^ effector-memory T cells (104, 105). The gene-expression analysis indicated that CD8^+^ effector-memory T cells were characterized by a mixed signature of T cell dysfunction, activation, effector function (106). Upon *in vitro* stimulation, CD8^+^ T cells were able to elicit a series of immune reactions including proliferation, degranulation, and production of IFN-γ, TNF-α, perforin, and granzyme B. Thereby, CD8^+^ decidual T cells restrict the activation to avoid rejecting the fetus but retain the immune defense against infections (106, 107).

The dominant proportion of CD8^+^ decidual effector-memory T cells suggests that antigens have been presented to CD8^+^ decidual T cells, which can elicit antigen-specific CD8^+^ T cell responses. Indeed, analysis of the TCR repertoires of effector-memory CD8^+^ T cells revealed that a few TCRβ were clonally expanded (108). Nevertheless, the antigens targeted by CD8^+^ decidual T cells are not fully understood and candidate antigens may be HLA-C, minor histocompatibility antigens (mHags), and pathogen-derived antigens (104).

The limited cytotoxicity of CD8^+^ decidual T cells can be attributed to high expression of a variety of immune checkpoint molecules including PD-1, TIM-3, LAG-3, and CTLA-4 under a decidual-specific immune microenvironment (105, 106, 108). The PD-1^+^TIM-3^+^CD8^+^ decidual T subset possessed increased proliferation potential and produced Th2 cytokines, exhibiting tolerant capacity toward trophoblasts, whereas trophoblasts in turn enriched PD1^+^TIM-3^+^CD8^+^ T cells in an HLA-C-dependent manner (109). Furthermore, PD-L1, the ligand for PD-1, has been found to be highly expressed on STBs, moderately expressed on intermediate trophoblasts, and barely expressed on CTB (110, 111). Both of STBs and intermediate trophoblasts come into contact with DICs including CD8^+^ T cells. The PD-L1/PD-1 interaction between trophoblasts and CD8^+^ T cells gives rise to an inhibitory signal, contributing to inhibition of CD8^+^ T cells cytotoxicity and maintenance of immunotolerance (Figure 5).

## Immunotherapy of RSA

As mentioned earlier, the establishment and maintenance of maternal–fetal immune tolerance depends on the harmonious dialog between trophoblasts and DICs. Abnormal mother–fetal dialog will lead to dysfunction of maternal–fetal immunotolerance and thus bring about pregnancy loss and pregnancy complications.

The research on maternal–fetal immunotolerance is not only to analyze the immunological truth behind successful pregnancy, but more importantly, it is expected to provide a new theoretical basis for the diagnosis and treatment of clinical pregnancy-related complications caused by immune tolerance disorders including RSA. RSA has become one of the most frustrating and difficult problems in reproductive medicine and puts a huge burden on patients and their families.

Indeed, immunotolerance of pregnancy is similar to tumor immune escape. Recently, immunotherapy has developed rapidly; in particular, anti-tumor therapy has achieved remarkable achievements. Therefore, the application of immunotherapy in RSA is also worthy of attention and exploration. The immunotherapy of RSA is summarized as follows.

### Controversial Immunotherapies of RSA

Lymphocyte active immunotherapy (LIT) for pregnancy preservation is a process in which peripheral white blood cells, collected from the husband or a third person, are injected into the prospective mother to prepare her immune system for tolerating the embryo's antigens (112). However, a lot of research advocated that the efficiency of LIT for the treatment of RSA is inconsistent and controversial (113, 114).

Intravenous immunoglobulin (IVIg) passive immunotherapy is another approach widely used in many areas of the world. On the basis of the hypothesis that RSA was associated with a lack of blocking antibodies normally produced by the mother's immune system to protect the fetus against immunologic rejections, IVIg treatment was attempted as a novel therapeutic approach for unexplained RSA (URSA) as early as 1989 (115). Although some randomized clinical trials designed to evaluate IVIg therapeutic efficacy in women with unexplained RSA have shown that IVIg is supposed to be more advantageous than LIT (115–117), others have come up with the opposite conclusion that evidence is insufficient to support the beneficial effects of IVIg on an unexplained RSA (118–120).

### Promising Strategy for Immunotherapy of RSA

Given the inconsistent and controversial efficacy of current immunotherapies such as LIT and IVIg, it is an urgent mission for reproductive immunologists to explore feasible immunotherapies. Tumor immunotherapy based on the tumor immune escape mechanism, has developed dramatically. In particular, the use of immune checkpoint inhibitors has become standard therapy in many tumors. The breakthrough in anti-tumor immunotherapy brings great confidence and new ideas to the field of reproductive immunology, since reproductive immunity and tumor immunity display many similarities. Moreover, the roles of immune checkpoints such as CTLA4, PD-1, and TIM-3 in multiple critical processes during pregnancy have been gradually demonstrated by our team and other researchers. For example, the TIM-3 signal is involved in the functional regulation of NK cells, the PD-1 signal modulates the polarization of macrophages and the cytotoxicity of cytotoxic T cells, and CTLA-4 is an important effector molecule on the surface of Treg cells. Whether immune checkpoints could be the targeted immunotherapy for reproductive immune disorders such as RSA deserves to be further explored.

Besides the immunotherapy targeting checkpoints, the use of mesenchymal stem cells (MSCs) has been a novel promising therapeutic approach in many clinical conditions, such as tumor, autoimmune diseases, and inflammation-related diseases. Excitingly, our research team found that MSCs executed immunotherapeutic effect in both the LPS-induced abortion model and the immune response-mediated spontaneous abortion model via inhibition of CD4^+^ T cell proliferation and promotion of macrophage M2 polarization (52). Consistent results were obtained by other researches (121–125). Therefore, the prospect of MSCs as a potential therapeutic strategy for RSA is worth expecting and needs more clinical trials.

Far less mature than tumor immunotherapy, novel immunotherapeutic strategies for RSA is on the way, and much more effort should be paid by reproductive immunologists. All novel therapies are based on clear physiologic and pathologic mechanisms. Thus, focusing on the crosstalk of trophoblasts and DICs and their roles in normal pregnancy and RSA is indeed critical to revealing the maternal immune tolerance mechanism and paves the way for the promising immunotherapy of RSA.

## Conclusion and Perspectives

Maternal immune tolerance to the semi-allogeneic fetus is an important cornerstone for the smooth progress of pregnancy until successful delivery. Trophoblasts are the only fetal-derived cells that come into direct contact with the maternal immune system. Rather than rejecting the fetus, trophoblasts actively participate in establishing and maintaining immune tolerance through delicate dialogs with DICs. Abnormal conversation between trophoblasts and DICs will lead to dysregulated maternal–fetal immunity, which is supposed to be a potential cause of pregnancy complications including RSA. Therefore, further exploration of the interactive dialog between trophoblasts and DICs is expected to interpret the mechanism of pregnancy tolerance. More importantly, it will provide a new scientific basis for the diagnosis and treatment of disorders associated with pregnancy tolerance abnormalities. At the same time, the research breakthrough of maternal–fetal immune tolerance will also inspire and promote the research on tumor immune escape and transplantation immunity.

## Data Availability Statement

The original contributions presented in the study are included in the article/supplementary material, further inquiries can be directed to the corresponding author/s.

## Author Contributions

MD and DL are responsible for the conception and design of this review. LX and YL performed literature search, wrote the first draft of the manuscript and generated all the table and figures. All authors revised the manuscript and approved the submitted version.

## Conflict of Interest

The authors declare that the research was conducted in the absence of any commercial or financial relationships that could be construed as a potential conflict of interest.

## References

[1] Gellersen B Brosens JJ. Cyclic decidualization of the human endometrium in reproductive health and failure. Endocr Rev. (2014) 35:851–905. 10.1210/er.2014-1045 25141152

[2] Brosens I Puttemans P Benagiano G. Placental bed research: I. The placental bed: from spiral arteries remodeling to the great obstetrical syndromes. Am J Obstet Gynecol. (2019) 221:437–56. 10.1016/j.ajog.2019.05.044 31163132

[3] Billingham RE Brent L Medawar PB. Actively acquired tolerance of foreign cells. Nature. (1953) 172:603–6. 10.1038/172603a0 13099277

[4] Zhang J Mo HQ Tian FJ Zeng WH Liu XR Ma XL. EIF5A1 promotes trophoblast migration and invasion via ARAF-mediated activation of the integrin/ERK signaling pathway. Cell Death Dis. (2018) 9:926. 10.1038/s41419-018-0971-5 30206208 PMC6134074

[5] Ma XL Li X Tian FJ Zeng WH Zhang J Mo HQ. Upregulation of RND3 affects trophoblast proliferation, apoptosis, and migration at the maternal-fetal interface. Front Cell Dev Biol. (2020) 8:153. 10.3389/fcell.2020.00153 32232044 PMC7083256

[6] Mo HQ Tian FJ Ma XL Zhang YC Zhang CX Zeng WH. PDIA3 regulates trophoblast apoptosis and proliferation in preeclampsia via the MDM2/p53 pathway. Reproduction. (2020) 160:293–305. 10.1530/rep-20-0156 32585639

[7] Qin S Zhang Y Zhang J Tian F Sun L He X. SPRY4 regulates trophoblast proliferation and apoptosis via regulating IFN-γ-induced STAT1 expression and activation in recurrent miscarriage. Am J Reprod Immunol. (2020) 83:e13234. 10.1111/aji.13234 32196809

[8] Solano ME. Decidual immune cells: Guardians of human pregnancies. Best Pract Res Clin Obstet Gynaecol. (2019) 60:3–6. 10.1016/j.bpobgyn.2019.05.009 31285174

[9] Bulmer JN Williams PJ Lash GE. Immune cells in the placental bed. Int J Dev Biol. (2010) 54:281–94. 10.1387/ijdb.082763jb 19876837

[10] Faas MM De Vos P. Innate immune cells in the placental bed in healthy pregnancy and preeclampsia. Placenta. (2018) 69:125–33. 10.1016/j.placenta.2018.04.012 29748088

[11] Liu S Diao L Huang C Li Y Zeng Y Kwak-Kim JYH. The role of decidual immune cells on human pregnancy. J Reprod Immunol. (2017) 124:44–53. 10.1016/j.jri.2017.10.045 29055791

[12] Piliszek A Grabarek JB Frankenberg SR Plusa B. Cell fate in animal and human blastocysts and the determination of viability. Mol Hum Reprod. (2016) 22:681–90. 10.1093/molehr/gaw002 26769259

[13] Knöfler M Haider S Saleh L Pollheimer J Gamage T James J. Human placenta and trophoblast development: key molecular mechanisms and model systems. Cell Mol Life Sci. (2019) 76:3479–96. 10.1007/s00018-019-03104-6 31049600 PMC6697717

[14] Burton GJ Fowden AL. The placenta: a multifaceted, transient organ. Philos Trans R Soc Lond B Biol Sci. (2015) 370:20140066. 10.1098/rstb.2014.0066 25602070 PMC4305167

[15] Fisher SJ. Why is placentation abnormal in preeclampsia? Am J Obstet Gynecol. (2015) 213:S115–122. 10.1016/j.ajog.2015.08.042 26428489 PMC4592742

[16] Romero R Kusanovic JP Chaiworapongsa T Hassan SS. Placental bed disorders in preterm labor, preterm PROM, spontaneous abortion and abruptio placentae. Best Pract Res Clin Obstet Gynaecol. (2011) 25:313–27. 10.1016/j.bpobgyn.2011.02.006 21388889 PMC3092823

[17] Liu Y Fan X Wang R Lu X Dang YL Wang H. Single-cell RNA-seq reveals the diversity of trophoblast subtypes and patterns of differentiation in the human placenta. Cell Res. (2018) 28:819–32. 10.1038/s41422-018-0066-y 30042384 PMC6082907

[18] Vento-Tormo R Efremova M Botting RA Turco MY Vento-Tormo M Meyer KB. Single-cell reconstruction of the early maternal-fetal interface in humans. Nature. (2018) 563:347–53. 10.1038/s41586-018-0698-6 30429548 PMC7612850

[19] Moffett A Chazara O Colucci F. Maternal allo-recognition of the fetus. Fertil Steril. (2017) 107:1269–72. 10.1016/j.fertnstert.2017.05.001 28577615

[20] Krop J Heidt S Claas FHJ Eikmans M. Regulatory T cells in pregnancy: it is not all about FoxP3. Front Immunol. (2020) 11:1182. 10.3389/fimmu.2020.01182 32655556 PMC7324675

[21] Yang F Zheng Q Jin L. Dynamic function and composition changes of immune cells during normal and pathological pregnancy at the maternal-fetal interface. Front Immunol. (2019) 10:2317. 10.3389/fimmu.2019.02317 31681264 PMC6813251

[22] Fu B Wei H. Decidual natural killer cells and the immune microenvironment at the maternal-fetal interface. Sci China Life Sci. (2016) 59:1224–31. 10.1007/s11427-016-0337-1 27905000

[23] Vacca P Chiossone L Mingari MC Moretta L. Heterogeneity of NK cells and other innate lymphoid cells in human and murine decidua. Front Immunol. (2019) 10:170. 10.3389/fimmu.2019.00170 30800126 PMC6375891

[24] Guia S Jaeger BN Piatek S Mailfert S Trombik T Fenis A. Confinement of activating receptors at the plasma membrane controls natural killer cell tolerance. Sci Signal. (2011) 4:ra21. 10.1126/scisignal.2001608 21467299

[25] Gong H Chen Y Xu J Xie X Yu D Yang B. The regulation of ovary and conceptus on the uterine natural killer cells during early pregnancy. Reprod Biol Endocrinol. (2017) 15:73. 10.1186/s12958-017-0290-1 28874155 PMC5585937

[26] Parham P Moffett A. Variable NK cell receptors and their MHC class I ligands in immunity, reproduction and human evolution. Nat Rev Immunol. (2013) 13:133–44. 10.1038/nri3370 23334245 PMC3956658

[27] Long W Shi Z Fan S Liu L Lu Y Guo X. Association of maternal KIR and fetal HLA-C genes with the risk of preeclampsia in the Chinese Han population. Placenta. (2015) 36:433–7. 10.1016/j.placenta.2014.05.008 24951171

[28] Xiong S Sharkey AM Kennedy PR Gardner L Farrell LE Chazara O. Maternal uterine NK cell-activating receptor KIR2DS1 enhances placentation. J Clin Invest. (2013) 123:4264–72. 10.1172/jci68991 24091323 PMC4382274

[29] Kennedy PR Chazara O Gardner L Ivarsson MA Farrell LE Xiong S. Activating KIR2DS4 is expressed by uterine NK cells and contributes to successful pregnancy. J Immunol. (2016) 197:4292–300. 10.4049/jimmunol.1601279 27815424 PMC5114884

[30] Iwaszko M Bogunia-Kubik K. Clinical significance of the HLA-E and CD94/NKG2 interaction. Arch Immunol Ther Exp (Warsz). (2011) 59:353–67. 10.1007/s00005-011-0137-y 21800130

[31] Kanevskiy L Erokhina S Kobyzeva P Streltsova M Sapozhnikov A Kovalenko E. Dimorphism of HLA-E and its disease association. Int J Mol Sci. (2019) 20:496. 10.3390/ijms20215496 31690066 PMC6862560

[32] Bai Y Liang J Liu W Wang F Li C. Possible roles of HLA-G regulating immune cells in pregnancy and endometrial diseases via KIR2DL4. J Reprod Immunol. (2020) 142:103176. 10.1016/j.jri.2020.103176 32711226

[33] Nowak I Wilczyńska K Wilczyński JR Malinowski A Radwan P Radwan M. KIR, LILRB and their ligands' genes as potential biomarkers in recurrent implantation failure. Arch Immunol Ther Exp. (2017) 65:391–9. 10.1007/s00005-017-0474-6 28523429 PMC5602049

[34] Llano M Lee N Navarro F García P Albar JP Geraghty DE. HLA-E-bound peptides influence recognition by inhibitory and triggering CD94/NKG2 receptors: preferential response to an HLA-G-derived nonamer. Eur J Immunol. (1998) 28:2854–63. 10.1002/(sici)1521-4141(199809)28:09<2854::Aid-immu2854>3.0.Co;2-w 9754572

[35] Ferreira LMR Meissner TB Tilburgs T Strominger JL. HLA-G: at the interface of maternal-fetal tolerance. Trends Immunol. (2017) 38:272–86. 10.1016/j.it.2017.01.009 28279591

[36] Tilburgs T Evans JH Crespo ÂC Strominger JL. The HLA-G cycle provides for both NK tolerance and immunity at the maternal-fetal interface. Proc Natl Acad Sci USA. (2015) 112:13312–7. 10.1073/pnas.1517724112 26460007 PMC4629323

[37] Li YH Zhou WH Tao Y Wang SC Jiang YL Zhang D. The Galectin-9/Tim-3 pathway is involved in the regulation of NK cell function at the maternal-fetal interface in early pregnancy. Cell Mol Immunol. (2016) 13:73–81. 10.1038/cmi.2014.126 25578313 PMC4711677

[38] Tan HX Yang SL Li MQ Wang HY. Autophagy suppression of trophoblast cells induces pregnancy loss by activating decidual NK cytotoxicity and inhibiting trophoblast invasion. Cell Commun Signal. (2020) 18:73. 10.1186/s12964-020-00579-w 32398034 PMC7218578

[39] Wang XQ Zhou WJ Hou XX Fu Q Li DJ. Trophoblast-derived CXCL16 induces M2 macrophage polarization that in turn inactivates NK cells at the maternal-fetal interface. Cell Mol Immunol. (2018) 15:1038–46. 10.1038/s41423-018-0019-x 29588487 PMC6269500

[40] Huhn O Ivarsson MA Gardner L Hollinshead M Stinchcombe JC Chen P. Distinctive phenotypes and functions of innate lymphoid cells in human decidua during early pregnancy. Nat Commun. (2020) 11:381. 10.1038/s41467-019-14123-z 31959757 PMC6971012

[41] Murphy SP Fast LD Hanna NN Sharma S. Uterine NK cells mediate inflammation-induced fetal demise in IL-10-null mice. J Immunol. (2005) 175:4084–90. 10.4049/jimmunol.175.6.4084 16148158

[42] Murphy SP Hanna NN Fast LD Shaw SK Berg G Padbury JF. Evidence for participation of uterine natural killer cells in the mechanisms responsible for spontaneous preterm labor and delivery. Am J Obstet Gynecol. (2009) 200:308–9. 10.1016/j.ajog.2008.10.043 19114277 PMC3893044

[43] Thaxton JE Nevers T Lippe EO Blois SM Saito S Sharma S. NKG2D blockade inhibits poly(I:C)-triggered fetal loss in wild type but not in IL-10-/- mice. J Immunol. (2013) 190:3639–47. 10.4049/jimmunol.1203488 23455498 PMC3608719

[44] Geissmann F Manz MG Jung S Sieweke MH Merad M Ley K. Development of monocytes, macrophages, and dendritic cells. Science. (2010) 327:656–61. 10.1126/science.1178331 20133564 PMC2887389

[45] Wynn TA Chawla A Pollard JW. Macrophage biology in development, homeostasis and disease. Nature. (2013) 496:445–55. 10.1038/nature12034 23619691 PMC3725458

[46] Zhao Y Zou W Du J Zhao Y. The origins and homeostasis of monocytes and tissue-resident macrophages in physiological situation. J Cell Physiol. (2018) 233:6425–39. 10.1002/jcp.26461 29323706

[47] Shrivastava R Shukla N. Attributes of alternatively activated (M2) macrophages. Life Sci. (2019) 224:222–31. 10.1016/j.lfs.2019.03.062 30928403

[48] Zhou D Huang C Lin Z Zhan S Kong L Fang C. Macrophage polarization and function with emphasis on the evolving roles of coordinated regulation of cellular signaling pathways. Cell Signal. (2014) 26:192–7. 10.1016/j.cellsig.2013.11.004 24219909

[49] Tsao FY Wu MY Chang YL Wu CT Ho HN. M1 macrophages decrease in the deciduae from normal pregnancies but not from spontaneous abortions or unexplained recurrent spontaneous abortions. J Formos Med Assoc. (2018) 117:204–11. 10.1016/j.jfma.2017.03.011 28465068

[50] Yang SW Cho EH Choi SY Lee YK Park JH Kim MK. DC-SIGN expression in Hofbauer cells may play an important role in immune tolerance in fetal chorionic villi during the development of preeclampsia. J Reprod Immunol. (2017) 124:30–7. 10.1016/j.jri.2017.09.012 29049918

[51] Shimada S Ebina Y Iijima N Deguchi M Yamada H. Decidual CD68(+) HLA-DR(+) CD163(-) M1 macrophages increase in miscarriages with normal fetal chromosome. Am J Reprod Immunol. (2018) 79:91. 10.1111/aji.12791 29197148

[52] Li Y Zhang D Xu L Dong L Zheng J Lin Y. Cell-cell contact with proinflammatory macrophages enhances the immunotherapeutic effect of mesenchymal stem cells in two abortion models. Cell Mol Immunol. (2019) 16:908–20. 10.1038/s41423-019-0204-6 30778166 PMC6884632

[53] Chavan AR Griffith OW Wagner GP. The inflammation paradox in the evolution of mammalian pregnancy: turning a foe into a friend. Curr Opin Genet Dev. (2017) 47:24–32. 10.1016/j.gde.2017.08.004 28850905

[54] Abumaree MH Chamley LW Badri M El-Muzaini MF. Trophoblast debris modulates the expression of immune proteins in macrophages: a key to maternal tolerance of the fetal allograft? J Reprod Immunol. (2012) 94:131–41. 10.1016/j.jri.2012.03.488 22542910

[55] Huang Y Zhu XY Du MR Li DJ. Human trophoblasts recruited T lymphocytes and monocytes into decidua by secretion of chemokine CXCL16 and interaction with CXCR6 in the first-trimester pregnancy. J Immunol. (2008) 180:2367–75. 10.4049/jimmunol.180.4.2367 18250446

[56] Meng YH Zhou WJ Jin LP Liu LB Chang KK Mei J. RANKL-mediated harmonious dialogue between fetus and mother guarantees smooth gestation by inducing decidual M2 macrophage polarization. Cell Death Dis. (2017) 8:e3105. 10.1038/cddis.2017.505 29022922 PMC5682671

[57] Svensson-Arvelund J Mehta RB Lindau R Mirrasekhian E Rodriguez-Martinez H Berg G. The human fetal placenta promotes tolerance against the semiallogeneic fetus by inducing regulatory T cells and homeostatic M2 macrophages. J Immunol. (2015) 194:1534–44. 10.4049/jimmunol.1401536 25560409

[58] Cheng SB Sharma S. Interleukin-10: a pleiotropic regulator in pregnancy. Am J Reprod Immunol. (2015) 73:487–500. 10.1111/aji.12329 25269386 PMC4382460

[59] Thaxton JE Romero R Sharma S. TLR9 activation coupled to IL-10 deficiency induces adverse pregnancy outcomes. J Immunol. (2009) 183:1144–54. 10.4049/jimmunol.0900788 19561095 PMC2785500

[60] Hanna N Bonifacio L Weinberger B Reddy P Murphy S Romero R. Evidence for interleukin-10-mediated inhibition of cyclo- oxygenase-2 expression and prostaglandin production in preterm human placenta. Am J Reprod Immunol. (2006) 55:19–27. 10.1111/j.1600-0897.2005.00342.x 16364008

[61] Matthiesen L Berg G Ernerudh J Ekerfelt C Jonsson Y Sharma S. Immunology of preeclampsia. Chem Immunol Allergy. (2005) 89:49–61. 10.1159/000087912 16129952

[62] Lindau R Mehta RB Lash GE Papapavlou G Boij R Berg G. Interleukin-34 is present at the fetal-maternal interface and induces immunoregulatory macrophages of a decidual phenotype *in vitro*. Hum Reprod. (2018) 33:588–99. 10.1093/humrep/dey037 29579271

[63] Ding J Yang C Cheng Y Wang J Zhang S Yan S. Trophoblast-derived IL-6 serves as an important factor for normal pregnancy by activating Stat3-mediated M2 macrophages polarization. Int Immunopharmacol. (2020) 2020:106788. 10.1016/j.intimp.2020.106788 32718866

[64] Wang S Sun F Han M Liu Y Zou Q Wang F. Trophoblast-derived hyaluronan promotes the regulatory phenotype of decidual macrophages. Reproduction. (2019) 157:189–98. 10.1530/rep-18-0450 30605433

[65] Zhang Y Ma L Hu X Ji J Mor G Liao A. The role of the PD-1/PD-L1 axis in macrophage differentiation and function during pregnancy. Hum Reprod. (2019) 34:25–36. 10.1093/humrep/dey347 30500923

[66] Enninga EAL Harrington SM Creedon DJ Ruano R Markovic SN Dong H. Immune checkpoint molecules soluble program death ligand 1 and galectin-9 are increased in pregnancy. Am J Reprod Immunol. (2018) 79:95. 10.1111/aji.12795 29205636 PMC5814874

[67] Okuyama M Mezawa H Kawai T Urashima M. Elevated soluble PD-L1 in pregnant women's serum suppresses the immune reaction. Front Immunol. (2019) 10:86. 10.3389/fimmu.2019.00086 30833943 PMC6387906

[68] Zhang YH Aldo P You Y Ding J Kaislasuo J Petersen JF. Trophoblast-secreted soluble-PD-L1 modulates macrophage polarization and function. J Leukoc Biol. (2020) 108:983–98. 10.1002/jlb.1a0420-012rr 32386458 PMC8190653

[69] Lee CL Guo Y So KH Vijayan M Guo Y Wong VH. Soluble human leukocyte antigen G5 polarizes differentiation of macrophages toward a decidual macrophage-like phenotype. Hum Reprod. (2015) 30:2263–74. 10.1093/humrep/dev196 26307092

[70] Lee SK Kim JY Hur SE Kim CJ Na BJ Lee M. An imbalance in interleukin-17-producing T and Foxp3+ regulatory T cells in women with idiopathic recurrent pregnancy loss. Hum Reprod. (2011) 26:2964–71. 10.1093/humrep/der301 21926059

[71] Darmochwal-Kolarz D Kludka-Sternik M Tabarkiewicz J Kolarz B Rolinski J Leszczynska-Gorzelak B. The predominance of Th17 lymphocytes and decreased number and function of Treg cells in preeclampsia. J Reprod Immunol. (2012) 93:75–81. 10.1016/j.jri.2012.01.006 22370101

[72] Care AS Bourque SL Morton JS Hjartarson EP Robertson SA Davidge ST. Reduction in regulatory T cells in early pregnancy causes uterine artery dysfunction in mice. Hypertension. (2018) 72:177–87. 10.1161/hypertensionaha.118.10858 29785960

[73] Zeng W Liu Z Liu X Zhang S Khanniche A Zheng Y. Distinct Transcriptional and Alternative Splicing Signatures of Decidual CD4(+) T Cells in Early Human Pregnancy. Front Immunol. (2017) 8:682. 10.3389/fimmu.2017.00682 28659920 PMC5466981

[74] Saito S Nakashima A Shima T Ito M. Th1/Th2/Th17 and regulatory T-cell paradigm in pregnancy. Am J Reprod Immunol. (2010) 63:601–10. 10.1111/j.1600-0897.2010.00852.x 20455873

[75] Figueiredo AS Schumacher A. The T helper type 17/regulatory T cell paradigm in pregnancy. Immunology. (2016) 148:13–21. 10.1111/imm.12595 26855005 PMC4819144

[76] Nancy P Erlebacher A. T cell behavior at the maternal-fetal interface. Int J Dev Biol. (2014) 58:189–98. 10.1387/ijdb.140054ae 25023685 PMC4212519

[77] Liu F Guo J Tian T Wang H Dong F Huang H. Placental trophoblasts shifted Th1/Th2 balance toward Th2 and inhibited Th17 immunity at fetomaternal interface. Apmis. (2011) 119:597–604. 10.1111/j.1600-0463.2011.02774.x 21851417

[78] Munn DH Zhou M Attwood JT Bondarev I Conway SJ Marshall B. Prevention of allogeneic fetal rejection by tryptophan catabolism. Science. (1998) 281:1191–3. 10.1126/science.281.5380.1191 9712583

[79] Lee GK Park HJ Macleod M Chandler P Munn DH Mellor AL. Tryptophan deprivation sensitizes activated T cells to apoptosis prior to cell division. Immunology. (2002) 107:452–60. 10.1046/j.1365-2567.2002.01526.x 12460190 PMC1782830

[80] Guo PF Du MR Wu HX Lin Y Jin LP Li DJ. Thymic stromal lymphopoietin from trophoblasts induces dendritic cell-mediated regulatory TH2 bias in the decidua during early gestation in humans. Blood. (2010) 116:2061–9. 10.1182/blood-2009-11-252940 20538796

[81] Lin Y Zhong Y Shen W Chen Y Shi J Di J. TSLP-induced placental DC activation and IL-10(+) NK cell expansion: comparative study based on BALB/c x C57BL/6 and NOD/SCID x C57BL/6 pregnant models. Clin Immunol. (2008) 126:104–17. 10.1016/j.clim.2007.09.006 17974483

[82] Yokosuka T Kobayashi W Takamatsu M Sakata-Sogawa K Zeng H Hashimoto-Tane A. Spatiotemporal basis of CTLA-4 costimulatory molecule-mediated negative regulation of T cell activation. Immunity. (2010) 33:326–39. 10.1016/j.immuni.2010.09.006 20870175

[83] Sansom DM Walker LS. The role of CD28 and cytotoxic T-lymphocyte antigen-4 (CTLA-4) in regulatory T-cell biology. Immunol Rev. (2006) 212:131–48. 10.1111/j.0105-2896.2006.00419.x 16903911

[84] Chinen T Kannan AK Levine AG Fan X Klein U Zheng Y. An essential role for the IL-2 receptor in T(reg) cell function. Nat Immunol. (2016) 17:1322–33. 10.1038/ni.3540 27595233 PMC5071159

[85] Pandiyan P Zheng L Ishihara S Reed J Lenardo MJ. CD4+CD25+Foxp3+ regulatory T cells induce cytokine deprivation-mediated apoptosis of effector CD4+ T cells. Nat Immunol. (2007) 8:1353–62. 10.1038/ni1536 17982458

[86] Cheng G Yu A Malek TR. T-cell tolerance and the multi-functional role of IL-2R signaling in T-regulatory cells. Immunol Rev. (2011) 241:63–76. 10.1111/j.1600-065X.2011.01004.x 21488890 PMC3101713

[87] Sawant DV Yano H Chikina M Zhang Q Liao M Liu C. Adaptive plasticity of IL-10(+) and IL-35(+) T(reg) cells cooperatively promotes tumor T cell exhaustion. Nat Immunol. (2019) 20:724–35. 10.1038/s41590-019-0346-9 30936494 PMC6531353

[88] Sawant DV Hamilton K Vignali DA. Interleukin-35: expanding its job profile. J Interferon Cytokine Res. (2015) 35:499–512. 10.1089/jir.2015.0015 25919641 PMC4507123

[89] Chen W Jin W Hardegen N Lei KJ Li L Marinos N. Conversion of peripheral CD4+CD25- naive T cells to CD4+CD25+ regulatory T cells by TGF-beta induction of transcription factor Foxp3. J Exp Med. (2003) 198:1875–86. 10.1084/jem.20030152 14676299 PMC2194145

[90] Diefenhardt P Nosko A Kluger MA Richter JV Wegscheid C Kobayashi Y. IL-10 receptor signaling empowers regulatory T cells to control th17 responses and protect from GN. J Am Soc Nephrol. (2018) 29:1825–37. 10.1681/asn.2017091044 29866800 PMC6050938

[91] Collison LW Chaturvedi V Henderson AL Giacomin PR Guy C Bankoti J. IL-35-mediated induction of a potent regulatory T cell population. Nat Immunol. (2010) 11:1093–101. 10.1038/ni.1952 20953201 PMC3008395

[92] Plitas G Rudensky AY. Regulatory T cells: differentiation and function. Cancer Immunol Res. (2016) 4:721–5. 10.1158/2326-6066.Cir-16-0193 27590281 PMC5026325

[93] Samstein RM Josefowicz SZ Arvey A Treuting PM Rudensky AY. Extrathymic generation of regulatory T cells in placental mammals mitigates maternal-fetal conflict. Cell. (2012) 150:29–38. 10.1016/j.cell.2012.05.031 22770213 PMC3422629

[94] Sharma S. Natural killer cells and regulatory T cells in early pregnancy loss. Int J Dev Biol. (2014) 58:219–29. 10.1387/ijdb.140109ss 25023688 PMC4306453

[95] Paolino M Koglgruber R Cronin SJF Uribesalgo I Rauscher E Harreiter J. RANK links thymic regulatory T cells to fetal loss and gestational diabetes in pregnancy. Nature. (2021) 589:442–7. 10.1038/s41586-020-03071-0 33361811 PMC7116618

[96] Ramhorst R Fraccaroli L Aldo P Alvero AB Cardenas I Leirós CP. Modulation and recruitment of inducible regulatory T cells by first trimester trophoblast cells. Am J Reprod Immunol. (2012) 67:17–27. 10.1111/j.1600-0897.2011.01056.x 21819477 PMC3703637

[97] Tilburgs T Crespo ÂC van der Zwan A Rybalov B Raj T Stranger B. Human HLA-G+ extravillous trophoblasts: Immune-activating cells that interact with decidual leukocytes. Proc Natl Acad Sci USA. (2015) 112:7219–24. 10.1073/pnas.1507977112 26015573 PMC4466754

[98] Ma Y Yang Q Fan M Zhang L Gu Y Jia W. Placental endovascular extravillous trophoblasts (enEVTs) educate maternal T-cell differentiation along the maternal-placental circulation. Cell Prolif. (2020) 53:e12802. 10.1111/cpr.12802 32291850 PMC7260064

[99] Liu J Hao S Chen X Zhao H Du L Ren H. Human placental trophoblast cells contribute to maternal-fetal tolerance through expressing IL-35 and mediating iT(R)35 conversion. Nat Commun. (2019) 10:4601. 10.1038/s41467-019-12484-z 31601798 PMC6787064

[100] Du MR Guo PF Piao HL Wang SC Sun C Jin LP. Embryonic trophoblasts induce decidual regulatory T cell differentiation and maternal-fetal tolerance through thymic stromal lymphopoietin instructing dendritic cells. J Immunol. (2014) 192:1502–11. 10.4049/jimmunol.1203425 24453244 PMC3918863

[101] Ding H Dai Y Lei Y Wang Z Liu D Li R. Upregulation of CD81 in trophoblasts induces an imbalance of Treg/Th17 cells by promoting IL-6 expression in preeclampsia. Cell Mol Immunol. (2019) 16:302–12. 10.1038/s41423-018-0186-9 30487550 PMC6318306

[102] Tilburgs T Strominger JL. CD8+ effector T cells at the fetal-maternal interface, balancing fetal tolerance and antiviral immunity. Am J Reprod Immunol. (2013) 69:395–407. 10.1111/aji.12094 23432707 PMC3711858

[103] Zeng W Liu X Liu Z Zheng Y Yu T Fu S. Deep surveying of the transcriptional and alternative splicing signatures for decidual CD8(+) T cells at the first trimester of human healthy pregn ancy. Front Immunol. (2018) 9:937. 10.3389/fimmu.2018.00937 29780389 PMC5946033

[104] Tilburgs T Schonkeren D Eikmans M Nagtzaam NM Datema G Swings GM. Human decidual tissue contains differentiated CD8+ effector-memory T cells with unique properties. J Immunol. (2010) 185:4470–7. 10.4049/jimmunol.0903597 20817873

[105] Liu L Huang X Xu C Chen C Zhao W Li D. Decidual CD8(+)T cells exhibit both residency and tolerance signatures modulated by decidual stromal cells. J Transl Med. (2020) 18:221. 10.1186/s12967-020-02371-3 32487187 PMC7268777

[106] van der Zwan A Bi K Norwitz ER Crespo Â C Claas FHJ Strominger JL. Mixed signature of activation and dysfunction allows human decidual CD8(+) T cells to provide both tolerance and immunity. Proc Natl Acad Sci USA. (2018) 115:385–90. 10.1073/pnas.1713957115 29259116 PMC5777048

[107] Papúchová H Meissner TB Li Q Strominger JL Tilburgs T. The dual role of HLA-C in tolerance and immunity at the maternal-fetal interface. Front Immunol. (2019) 10:2730. 10.3389/fimmu.2019.02730 31921098 PMC6913657

[108] Morita K Tsuda S Kobayashi E Hamana H Tsuda K Shima T. Analysis of TCR repertoire and PD-1 expression in decidual and peripheral CD8(+) T cells reveals distinct immune mechanisms in miscarriage and preeclampsia. Front Immunol. (2020) 11:1082. 10.3389/fimmu.2020.01082 32582176 PMC7283903

[109] Wang SC Li YH Piao HL Hong XW Zhang D Xu YY. PD-1 and Tim-3 pathways are associated with regulatory CD8+ T-cell function in decidua and maintenance of normal pregnancy. Cell Death Dis. (2015) 6:e1738. 10.1038/cddis.2015.112 25950468 PMC4669692

[110] Veras E Kurman RJ Wang TL Shih IM. PD-L1 expression in human placentas and gestational trophoblastic diseases. Int J Gynecol Pathol. (2017) 36:146–53. 10.1097/pgp.0000000000000305 27362903 PMC5518625

[111] Lu B Teng X Fu G Bao L Tang J Shi H. Analysis of PD-L1 expression in trophoblastic tissues and tumors. Hum Pathol. (2019) 84:202–12. 10.1016/j.humpath.2018.10.001 30339966

[112] Hajipour H Nejabati HR Latifi Z Hamdi K Bahrami-Asl Z Fattahi A. Lymphocytes immunotherapy for preserving pregnancy: mechanisms and challenges. Am J Reprod Immunol. (2018) 80:e12853. 10.1111/aji.12853 29603821

[113] Jafarpour R Pashangzadeh S Mehdizadeh S Bayatipoor H Shojaei Z Motallebnezhad M. Functional significance of lymphocytes in pregnancy and lymphocyte immunotherapy in infertility: A comprehensive review and update. Int Immunopharmacol. (2020) 87:106776. 10.1016/j.intimp.2020.106776 32682255

[114] Achilli C Duran-Retamal M Saab W Serhal P Seshadri S. The role of immunotherapy in *in vitro* fertilization and recurrent pregnancy loss: a systematic review and meta-analysis. Fertil Steril. (2018) 110:1089–100. 10.1016/j.fertnstert.2018.07.004 30396553

[115] Mueller-Eckhardt G Heine O Neppert J Künzel W Mueller-Eckhardt C. Prevention of recurrent spontaneous abortion by intravenous immunoglobulin. Vox Sang. (1989) 56:151–4. 10.1111/j.1423-0410.1989.tb02018.x 2728393

[116] Mueller-Eckhardt G Heine O Polten B. IVIG to prevent recurrent spontaneous abortion. Lancet. (1991) 337:424–5. 10.1016/0140-6736(91)91197-3 1671439

[117] Yamada H Takeda M Maezawa Y Ebina Y Hazama R Tanimura K. A high dose intravenous immunoglobulin therapy for women with four or more recurrent spontaneous abortions. ISRN Obstet Gynecol. (2012) 2012:512732. 10.5402/2012/512732 22997588 PMC3446652

[118] Wang SW Zhong SY Lou LJ Hu ZF Sun HY Zhu HY. The effect of intravenous immunoglobulin passive immunotherapy on unexplained recurrent spontaneous abortion: a meta-analysis. Reprod Biomed Online. (2016) 33:720–736. 10.1016/j.rbmo.2016.08.025 27720163

[119] Christiansen OB Larsen EC Egerup P Lunoee L Egestad L Nielsen HS. Intravenous immunoglobulin treatment for secondary recurrent miscarriage: a randomised, double-blind, placebo-controlled trial. Bjog. (2015) 122:500–8. 10.1111/1471-0528.13192 25412569

[120] Stephenson MD Kutteh WH Purkiss S Librach C Schultz P Houlihan E. Intravenous immunoglobulin and idiopathic secondary recurrent miscarriage: a multicentered randomized placebo-controlled trial. Hum Reprod. (2010) 25:2203–9. 10.1093/humrep/deq179 20634190 PMC2923000

[121] Meng YH Zhu XH Yan LY Zhang Y Jin HY Xia X. Bone mesenchymal stem cells improve pregnancy outcome by inducing maternal tolerance to the allogeneic fetus in abortion-prone matings in mouse. Placenta. (2016) 47:29–36. 10.1016/j.placenta.2016.08.089 27780537

[122] Salek Farrokhi A Zarnani AH Moazzeni SM. Mesenchymal stem cells therapy protects fetuses from resorption and induces Th2 type cytokines profile in abortion prone mouse model. Transpl Immunol. (2018) 47:26–31. 10.1016/j.trim.2018.01.002 29317300

[123] Eskandarian M Moazzeni SM. Uterine dendritic cells modulation by mesenchymal stem cells provides a protective microenvironment at the feto-maternal interface: improved pregnancy outcome in abortion-prone mice. Cell J. (2019) 21:274–80. 10.22074/cellj.2019.6239 31210433 PMC6582417

[124] Muñoz-Fernández R De La Mata C Requena F Martín F Fernandez-Rubio P Llorca T. Human predecidual stromal cells are mesenchymal stromal/stem cells and have a therapeutic effect in an immune-based mouse model of recurrent spontaneous abortion. Stem Cell Res Ther. (2019) 10:177. 10.1186/s13287-019-1284-z 31200769 PMC6567662

[125] Sadighi-Moghaddam B Salek Farrokhi A Namdar Ahmadabad H Barati M Moazzeni SM. Mesenchymal stem cell therapy prevents abortion in CBA/J × DBA/2 mating. Reprod Sci. (2018) 25:1261–9. 10.1177/1933719117737848 29187052

